# Matching barriers and facilitators to implementation strategies: recommendations for community settings

**DOI:** 10.1186/s43058-023-00532-1

**Published:** 2023-11-21

**Authors:** Laura E. Balis, Bailey Houghtaling

**Affiliations:** 1 Gretchen Swanson Center for Nutrition, Omaha, NE 68514 USA; 2 Department of Human Nutrition, Virginia Tech, Foods, and Exercise, Blacksburg, VA 24061 USA; 3 School of Nutrition and Food Sciences, Louisiana State University (LSU) & LSU Agricultural Center, Baton Rouge, LA 70803 USA

**Keywords:** Implementation strategies, Community settings, Contextual factors, Pragmatism, Primary prevention, Public health

## Abstract

**Background:**

Implementation science aims to improve the integration of evidence-based interventions in real-world settings. While its methods and models could potentially apply to any field with evidence-based interventions, most research thus far has originated in clinical settings. Community settings often have fewer resources, missions beyond health, and a lack of support and expertise to implement evidence-based interventions when compared to many clinical settings. Thus, selecting and tailoring implementation strategies in community settings is particularly challenging, as existing compilations are primarily operationalized through clinical setting terminology. In this debate, we (1) share the process of using an existing match tool to select implementation strategies to increase uptake of nutrition and physical activity policy, systems, and environment interventions in community settings and (2) discuss the challenges of this process to argue that selecting implementation strategies in community settings has limited transferability from clinical settings and may require a unique implementation strategy compilation and pragmatic matching tool.

**Matching barriers to implementation strategies:**

The impetus for this debate paper came from our work selecting implementation strategies to improve the implementation and eventual scaling of nutrition and physical activity policy, systems, and environment interventions in a community settings. We conducted focus groups with practitioners and used the Consolidated Framework for Implementation Research-Expert Recommendations for Implementing Change match tool to select potential implementation strategies to overcome prominent barriers. There was limited congruence between tool outputs and optimal strategies, which may in part be due to differences in context between clinical and community settings. Based on this, we outline needs and recommendations for developing a novel and pragmatic matching tool for researchers and practitioners in community settings.

**Conclusions:**

More work is needed to refine the implementation barrier-strategy matching process to ensure it is relevant, rapid, and rigorous. As leading implementation strategy scholars note, as more researchers document contextual factors and strategies selected to address them, the knowledge base will increase, and refined mapping processes can emerge.

Contributions to the literature

•Implementation science methods and models could be broadly applied to settings outside of health services research, but terminology and transferability are challenges
•Suggested methods for matching implementation determinants to strategies are resource intensive and less feasible in community public health settings.
•We argue that due to differences between settings and the need for pragmatic, practitioner-friendly tools, a new implementation strategy compilation and matching tool for community settings is needed

## Background

Implementation science seeks to understand and improve the integration of evidence-based interventions (EBIs) within specific settings [1, 2]. Thus, by definition, implementation science applies to any field with EBIs and real-world settings in which to apply them. Although implementation science has roots in multiple disciplines from agriculture to public policy [3, 4], current research on implementation methods, models, and measures primarily originated in clinical settings [5–9]. This has resulted in valuable contributions to the field, including the establishment of taxonomies of implementation strategies and guidance on implementation outcomes [5, 7]. However, it is challenging to apply this research to settings (e.g., community-based public health) outside of clinical health services [10–12].

Consider the differences between clinical and community settings. Community practice settings implement chronic disease prevention interventions where people live, work, and play [11–14]. These settings are often challenged with fewer resources (e.g., staff and funding) for program implementation [11, 15]. As well, community-based organizations and the settings they deliver EBI in typically have missions beyond public health (e.g., a primary focus on youth development, education, or retail sales and profit) [11, 16, 17], which can result in a mismatch between EBIs and organizational missions [15]. Overall, community-based organizations face a lack of support and expertise to implement EBIs [15] when compared to clinical settings. For example, implementation science is embedded within the Veterans Administration as part of the organization’s mission, with resources, funding, and personnel dedicated to implementation science [18, 19], while, to the best of our knowledge, an equivalent, robust structure does not exist in community settings.

Therefore, selecting and tailoring implementation strategies (i.e., methods or techniques used to improve the integration of research into practice settings) [20] in community settings can be particularly challenging [10]. Multiple compilations of implementation strategies exist, but are primarily operationalized through clinical setting terminology [5, 6, 9, 21], limiting the extent to which they apply to community settings [10, 22]. Selecting which implementation strategies to use to address implementation determinants – a key step in implementation research and practice [23] – is also challenging. Recommendations for linking implementation determinants to implementation strategies using theoretical or conceptual reasoning exist (e.g., concept mapping, group model building, conjoint analysis, implementation mapping, and causal pathway development), but typically require specific software, specialized statistical analysis, or lengthy processes that may not be accessible or feasible in community settings [22, 24, 25]. While these methods have been used in behavioral health settings (e.g., clinical practice with the Department of Veterans Affairs) [24], there is limited evidence of their use for selection and tailoring of implementation strategies in varied community settings [26–28].

One method of selecting and tailoring implementation strategies that may be more feasible for community settings is the Consolidated Framework for Implementation Research (CFIR)—Expert Recommendations for Implementing Change (ERIC) match tool [29]. CFIR is one of the most widely used determinant frameworks in implementation science [29, 30] and was developed to encompass several pre-existing implementation determinant frameworks [31]. It includes multiple domains (innovation, outer setting, inner setting, individuals, and implementation process), of which one or more can be selected for identifying contextual factors that influence or “determine” adoption, implementation, or maintenance [31, 32]. ERIC is a compilation of implementation strategies developed through a multi-phase Delphi process with experts in clinical implementation research [5]. The compilation serves as a useful starting point for selecting implementation strategies, provides common language and terminology for these strategies in clinical settings, and allows for systematic reporting and synthesis across studies to better understand which strategies are effective for which EBIs in which contexts [29]. The CFIR-ERIC match tool was developed by a panel of expert implementation science researchers and practitioners by ranking implementation strategies that best address barriers corresponding with each CFIR construct [29]. The tool is open-access and simple to use through imputing CFIR constructs identified as barriers and receiving an output of matched implementation strategies.

The purpose of this debate is twofold. First, we document the process of using the CFIR-ERIC match tool to select implementation strategies to increase uptake of nutrition and physical activity policy, systems, and environment (PSE) interventions in community settings. Second, we discuss limitations of this process to argue that selecting implementation strategies in community settings has limited transferability from clinical settings. A unique implementation strategy compilation and pragmatic matching tool that researchers and practitioners in community settings can easily use may be required.

### Matching barriers to implementation strategies: a community setting example

The impetus for this debate paper came from our work selecting implementation strategies to improve the implementation and eventual scaling of PSEs delivered through Louisiana Cooperative Extension Service (LCES) [33]. Extension is a national public health delivery system associated with US land grant universities that has recently been tasked with PSE change implementation [33–36]. Nutrition and physical activity PSE interventions implemented through Extension vary by community; examples include healthy food retail, complete streets policies, safe routes to school, workplace policies, and shared use agreements [37]. While there have been successes, PSE interventions are challenging to implement, and they have not yet penetrated the national Extension system [16, 38]. In our formative study, we assessed barriers and facilitators to PSE implementation in LCES using semi-structured focus groups with delivery agents serving rural Louisiana communities following the 2022 CFIR [31, 32]. Key findings revealed implementation barriers at multiple levels [33]. In particular, communications, access to knowledge and information, partnerships and connections, individuals’ opportunity and capability, and innovation complexity were mentioned most frequently [33].

We used the identified barriers as inputs to the CFIR-ERIC match tool and reviewed the resultant output of implementation strategies. However, it was difficult to determine which implementation strategies to select, given inconsistencies with our community setting. For example, “Build a coalition” is often already the starting point for community work, including for LCES delivery agents [33], and other highly ranked strategies have limited relevance to community practice amid limited resources (e.g., “Conduct small cyclical tests of change.”) Therefore, we chose to instead review the full output of ERIC implementation strategies in comparison to noted barriers to nutrition and physical activity PSE interventions within LCES [33] and independently selected and came to agreement on relevant strategies. Then, given similarity between certain implementation strategies, and the need for an abbreviated list suitable for LCES partner feedback, we used Leeman’s categories [20] as a guide to classify selected strategies and broadened some strategy descriptions to encompass similarities and decrease repetition. This resulted in a condensed list of implementation strategies relevant to addressing barriers to nutrition and physical activity PSE interventions in rural Louisiana for the purpose of gaining partner feedback about strategy importance and future tailoring needs (see Table 1). As one example of this process, a prominent barrier to PSE implementation identified through the formative work was Communication (the “Networks and Communication” construct in the original version of the CFIR, which is used in the CFIR-ERIC matching tool). The highest ranked implementation strategy to address this barrier was “Promote network weaving.” This was reviewed with other similar strategies (“Shadow other networks” and “Visit other sites”) and condensed into “Peer learning” to capture multiple methods of learn from others (e.g., learning collaboratives, shadowing other delivery agents, sharing success stories) that are more commonly used within community settings.

**Table 1:** Revised implementation strategies for integrating policy, systems, and environment interventions in a community setting

Revised strategy	Definition	Original ERIC strategies
Educate other groups	Hold meetings for different groups (e.g., policy makers, administrators, other staff, community members) to explain PSE changes and increase demand for them	Conduct educational meetings
Training	Interactive, ongoing training, including training-the-trainer strategies, to improve knowledge, skills, self-efficacy, and motivation	Conduct ongoing training
		Make training dynamic
		Use train the trainer strategies
Technical assistance	Offering of interactive problem-solving, technical assistance, and consultation support by local personnel or other experts	Provide local technical assistance
		Centralize technical assistance
		Facilitation
		Provide supervision
Peer learning	Share and learn from others through a learning collaborative, shadowing other Agents, sharing success stories, or by visiting other sites	Shadow other experts
		Visit other sites
		Promote network weaving
Start small	Use small PSE change pilots or temporary demonstration projects (e.g., pop-up event)	Stage implementation scale up
Develop and share a formal plan and terminology	Have a glossary of key PSE change terms with an implementation blueprint that includes goals and performance measures to share with implementers and community partners	Provide ongoing consultation
		Use an implementation adviser
		Develop an implementation glossary
		Develop a formal implementation blueprint
		Organize implementation team meetings
		Assess for readiness and identify barriers and facilitators
Identify and engage dedicated partners	Identify and leverage leaders in communities (e.g., PSE champions, early adopters)	Increase demand
		Create a learning collaborative
		Identify and prepare champions
		Identify early adopters
Strengthen existing processes	Build on existing procedures to strengthen approach, including assessing partners’ readiness, working with coalitions, and sharing PSE change resources	Build a coalition
		Capture and share local knowledge
		Develop resource sharing agreements
Increase access to new funding streams	Access new sources of funding for PSE changes such as from grants, contracts, or local government	Access new funding
		Fund and contract for the evidence-based program
Engage community members	Solicit feedback from community members and involve them in PSE planning and implementation	Involve priority population and support network
		Obtain and use priority population and support network feedback
Change organizational structures	Recruit and train leaders to prioritize and provide supervision in PSE changes, revise professional roles in Extension, and create incentive structures for PSE change facilitation	Alter incentive/allowance structures
		Recruit, designate and train for leadership
		Revise professional roles
		Create new delivery agent teams
		Mandate change
		Change record system
		Use capitated payments
Monitor and evaluate	Have access to tools and systems to monitor PSE change implementation to inform continuous quality improvement	Develop and implement tools for quality monitoring
		Develop and organize quality monitoring systems
		Purposely reexamine the implementation

Despite the promise of the CFIR-ERIC match tool, it did not work as an easy, accurate matching system for the PSE intervention-LCES context. First, we acknowledge that this may be due to known limitations of the matching tool. As the authors note, the tool should be used with caution given a lack of consensus in specific barrier-implementation strategy matches (i.e., there was much heterogeneity in participants’ responses to the barrier prompts during tool development) [29]. Thus, the tool output does not present one implementation strategy per CFIR construct; a list of potential strategies with percentages of agreement are provided [29]. This can make it difficult for tool users to decide how many and which implementation strategies to select to address barriers, as there are no specific recommendations such as using a certain number of output strategies or a recommended cutoff point regarding cumulative percent (i.e., the level of endorsement across CFIR barriers [29]). Second, we recognize that using the match tool is only the first step in selecting and tailoring implementation strategies, as it can be useful for narrowing down implementation strategy options to determine strategies for full consideration before using other methods (e.g., conjoint analysis) [24, 29]. However, in our case, the tool output was not useful for narrowing the list of relevant implementation strategies.

Some of the challenges with using the matching tool could also be due to the differences in context between clinical and community settings. Implementation determinants are often different in clinic and community settings and may add challenges to implementation strategy matching — even when using a broad determinant framework such as CFIR [31, 32]. For example, constructs within the CFIR inner setting domain differ between community and clinical settings, as the infrastructure, culture, and funding of community settings is either not solely or at all focused on delivering evidence-based public health interventions [11, 17]. As well, the outer setting of CFIR can differ between community and clinical settings, given the outer setting often consists of the communities in which interventions are implemented, which are heavily influenced by local politics and dynamics, for example [33]. Finally, the innovation domain of CFIR also differs, as community organizations are increasingly implementing policy and environment-level interventions in real-world settings to change complex social determinants of health such as food access and built environments [11, 37, 39].

Another challenge is the difference in feasibility and importance of implementation strategies [6] in community settings. For example, strategies such as “use capitated payments” or “create or change credentialing and/or licensure standards” may be less applicable for community settings, even with tailored definitions and examples [10]. Certain implementation strategies are already inherent to the process of implementation in community settings, such as coalition building example noted above, and are thus not relevant recommendations to improve implementation. As well, challenges using the match tool likely also reflect the fact that different strategies are needed to implement different EBIs [29], and primary prevention interventions delivered in community settings have different implementation processes than clinical interventions delivered in health care settings.

### New terminology and tools for community settings

To overcome these limitations and speed the translation of research to practice in community settings, we provide ideas and recommendations to inform the development and dissemination of a novel and pragmatic set of implementation strategies and accompanying matching tool. First, this tool should be accessible to practitioners overseeing the delivery of evidence-based interventions without access to research partnerships and resources. Community settings do not have the time, staffing, or implementation science expertise to engage in detailed processes of selecting and tailoring implementation strategies. For example, community settings (without research partners bringing in grant funding) usually do not have the resources to devote to reviewing all possible implementation strategies (e.g., up to 100 h [40]). Overall, the current recommended approaches to implementation strategy selection require practitioners to partner with researchers, which perpetuates researchers holding the “power” rather than stepping away and giving control back to communities [41–43]. Community practitioners need pragmatic, evidence-based tools to select implementation strategies to address barriers and capitalize on facilitators — whether or not researchers are involved with improving program implementation.

Community setting practitioners are already using implementation strategies and will continue to use them with or without research partnerships. Providing practitioners with a tool they could use to select implementation strategies could improve practice in community settings. Use of a broader range of implementation strategies that address multiple levels of contextual factors could provide more “practice-based evidence” to inform evidence-based practice [44]. Otherwise, those in community settings typically resort to “training and hoping” [45] or defer to other strategies targeting individuals’ knowledge, skills, and abilities (e.g., educational materials or outreach visits) [46], and may miss potentially effective strategies aimed at overcoming organization and system-level barriers. Further, without an easy-to-use compilation and match tool, these technical assistance and educational support efforts often get lumped together, disallowing a collective understanding which specific strategies are most effective.

Second, we recommend a future matching tool employ a strength-based approach. Public health literature recommends using a strength-based approach to assess both needs and resources [47–51]. For example, rather than conducting a “needs assessment,” researchers and practitioners are encouraged to conduct a “strength and needs assessment” to understand barriers and facilitators [47–51]. Yet, the current literature on matching contextual factors to implementation strategies is focused on overcoming barriers rather than building on facilitators [29, 52]. The Implementation Research Logic Model includes a recommendation to consider both barriers and facilitators under implementation determinants, but lacks specific guidance on how to integrate facilitators to select and tailor implementation strategies [23]. While overcoming barriers is a necessary and important goal, for community settings, there is a need to also incorporate the preference for capitalizing on facilitators.

Overall, there is a need for specific guidance on incorporating facilitators into implementation strategy selection and tailoring. This could include changing which strategies are selected to complement existing strengths within systems or providing recommendations to tailor implementation strategies to capitalize on facilitators. For example, working with community coalitions has been identified as both a barrier and facilitator to integrate nutrition and physical activity PSE interventions in community settings [33, 35, 36]. Thus, the strategy “build a coalition” could be tailored to “offer coalition coaching” to capitalize on the presence of community coalitions while addressing the barrier of challenges with coalition processes [36].

Third, we recommend the new matching tool be developed for and by community setting practitioners and researchers across multiple settings. As recommended by implementation strategy experts, the tool should avoid jargon and embody real-world perspectives [53]. With diverse input, the tool could apply broadly to multiple community settings (from faith-based to education to social services or healthy food retail, for example) [11, 17] and patterns/practices (e.g., chronic disease primary prevention, including nutrition, physical activity, and tobacco use), and could be tailored for project-related needs for specific settings (e.g., Early Head Start as one educational setting). While the matching tool should be designed for ease of use by practitioners, its use by community-engaged researchers would also build the evidence base on for whom, under what conditions, and how [54] implementation strategies work in community settings. Indeed, developing a new compilation of implementation strategies for community settings is a focus area of the National Institute of Health’s Consortium for Cancer Implementation Science, with a funded project in progress to develop a public good to share this compilation [55].

## Conclusion

The goal of this debate was to share our approach to selecting implementation strategies in a community setting to inform pragmatic tools for researchers and practitioners. More work is needed to refine the implementation barrier-strategy matching process to ensure it is relevant, rapid, and rigorous for community public health work [56]. As leading implementation strategy scholars note, as more researchers document contextual factors and strategies selected to address them, the knowledge base will increase and a refined mapping processes can emerge [29].

## Data Availability

Data sharing is not applicable to this article as no datasets were generated or analyzed during the current study.

## Abbreviations

EBIs: Evidence-based interventions
CFIR: Consolidated Framework for Implementation Research
ERIC: Expert Recommendations for Implementing Change
PSE: Policy, systems, and environment
LCES: Louisiana Cooperative Extension Service
